# The interplay of suppressive soil bacteria and plant root exudates determines germination of microsclerotia of *Verticillium longisporum*

**DOI:** 10.1128/aem.00589-24

**Published:** 2024-05-30

**Authors:** Sarenqimuge Sarenqimuge, Yao Wang, Mohammad Alhussein, Birger Koopmann, Andreas von Tiedemann

**Affiliations:** 1 Plant Pathology and Plant Protection Division, Department of Crop Sciences, Faculty of Agriculture, Georg-August University Göttingen, Göttingen, Germany; 2 Agricultural Entomology Division, Department of Crop Sciences, Faculty of Agriculture, Georg-August University Göttingen, Göttingen, Germany; University of Tennessee at Knoxville, Knoxville, Tennessee, USA

**Keywords:** *Brassica napus*, soil-borne pathogen, fungus disease, soil microorganism, plant-microbe interaction

## Abstract

Dormant microsclerotia play a vital role in the survival and spread of *Verticillium longisporum*, as they can stay viable in the soil and maintain their infectivity for many years. In our previous work, we revealed that soil bacterial volatiles are a key inhibitory factor causing microsclerotia dormancy in the soil. In this study, we further demonstrate that root exudates collected from both host and non-host plants can effectively rescue microsclerotia from bacterial suppression and initiate germination. To identify the specific compounds in root exudates responsible for microsclerotia germination, we fractionated the collected root exudates into polar and non-polar compounds. Subsequently, we conducted comprehensive bioassays with each fraction on germination-suppressed microsclerotia. The result revealed a pivotal role of primary metabolites in root exudates, particularly glutamic acid, in triggering microsclerotia germination and overcoming bacterial inhibition. Moreover, our studies revealed a decrease in inhibitory bacterial volatile fatty acids when bacteria were cultured in the presence of root exudates or glutamic acid. This suggests a potential mechanism, by which root exudates set-off bacterial suppression on microsclerotia. Here, we reveal for the first time that plant root exudates, instead of directly inducing the germination of microsclerotia, enact a set-off effect by counteracting the suppressive impact of soil bacteria on the microsclerotia germination process. This nuanced interaction advances our understanding of the multifaceted dynamics governing microsclerotia dormancy and germination in the soil environment.

**IMPORTANCE:**

Our research provides first-time insights into the crucial interaction between plant root exudates and soil bacteria in regulating the germination of *Verticillium longisporum* microsclerotia, a significant structure in the survival and proliferation of this soil-borne pathogen. We describe so far unknown mechanisms, which are key to understand how root infections on oilseed rape can occur. By pinpointing primary metabolites in root exudates as key factors in overcoming bacteria-induced dormancy and promote microsclerotia germination, our study highlights the potential for exploiting plant - as well as soil microbe-derived - compounds to control *V. longisporum*. This work underscores the importance of elucidating the nuanced interactions within the soil ecosystem to devise innovative strategies for managing root infective plant diseases, thereby contributing to the resilience and health of cropping systems.

## INTRODUCTION

*Verticillium longisporum* is a soil-borne, root-infecting, vascular pathogen that poses a significant threat to brassicaceous crops. The disease starts from viable dormant microsclerotia, which are left behind in the soil after harvest. These small, melanized structures can remain viable in the soil for many years, thus acting as long-term inoculum reservoir enabling the pathogen to persist in the soil between cropping seasons. In order to initiate a successful root infection, microsclerotia must germinate when brassicaceous crops are planted in the field, which is a prerequisite to enter the host vascular system (1, 2).

Efforts to manage this pathogen and mitigate its impact on brassicaceous crops require a better understanding of the interactions among the pathogen, the host plant, and the environmental factors determining dormancy and germination of microsclerotia (3, 4). Our previous study revealed that dormancy of microsclerotia in the soil is primarily attributed to the presence of bacterial acidic volatiles (3). In contrast to these fungistatic effects in natural soil environments, the presence of host plants may apparently trigger the germination of dormant microsclerotia, thus initiating infection (1). However, the mechanisms, by which germination is triggered in the presence of host roots are still unclear, which highlights the importance of understanding the communication between host plants and this plant pathogen.

The interaction and communication between plants and soil microbes largely depend on the production and secretion of a diverse array of plant compounds, collectively referred to as root exudates. These exudates consist of a complex mixture of organic compounds, including sugars, amino acids, organic acids and secondary metabolites (5–8). In contrast to the role of root exudates in the communication between symbionts and plants, our knowledge about the interaction between root exudates and soil-borne plant pathogens is poor. A common view is that root exudates may directly activate dormant resting propagules of soil-borne pathogens, which then initiate root infection (9). For instance, peanut root exudates had a significant promoting effect on spore germination, sporulation, and mycelial growth of two soil-borne pathogens, *Fusarium oxysporum* and *Fusarium solani*, when compared to the control (10). Zhang et al. (11) identified four phenolic acids in cotton root exudates, which exhibited a stimulating effect on the germination of *Verticillium dahliae* spores when they were present in low concentrations. Ren et al. (12) reported that root exudates of rice contained p-coumaric acid, which inhibited *F. oxysporum* f. sp *niveum* (FON) spore germination and sporulation while watermelon roots secreted ferulic acid, which stimulated FON spore germination and sporulation. This variation in phenolic acid composition within root exudates may elucidate the mechanism, by which intercropping watermelon with aerobic rice mitigates wilt disease caused by FON.

All these studies collectively highlight the significance of root exudates in influencing soil-borne pathogens. However, it is important to note that these previous investigations have focused solely on direct effects of root exudates on pathogens under sterile conditions, neglecting the complex interplay of environmental factors, such as the impact of soil microorganisms in a non-sterile soil environment. To the best of our knowledge, information is lacking about how root exudates, in the presence of soil bacteria (SB), affect the germination of microsclerotia of *V. longisporum* in the soil. Therefore, this study aims to elucidate the role of root exudates in promoting microsclerotia germination in the soil and uncover the specific components and mechanisms underlying this stimulatory effect. More specifically, our objectives include exploration of the effect of root exudates from both host and non-host plants in stimulating the germination of dormant microsclerotia in the soil, taking into account the role of the soil microbial environment in the interaction. The second aim was to identify the specific compound(s) in root exudates involved in inducing germination of microsclerotia and finally, we sought to uncover the mechanism, by which root exudates, in conjunction with SB, may stimulate the germination of dormant microsclerotia. From gaining deeper insights into the factors and mechanisms influencing the pivotal stages in *V. longisporum* life cycle, we ultimately expect novel and valuable perspectives to foster sustainable practices of disease control.

## RESULTS

### *In vitro* bioassays with collected native root exudates

**FIG 1:**
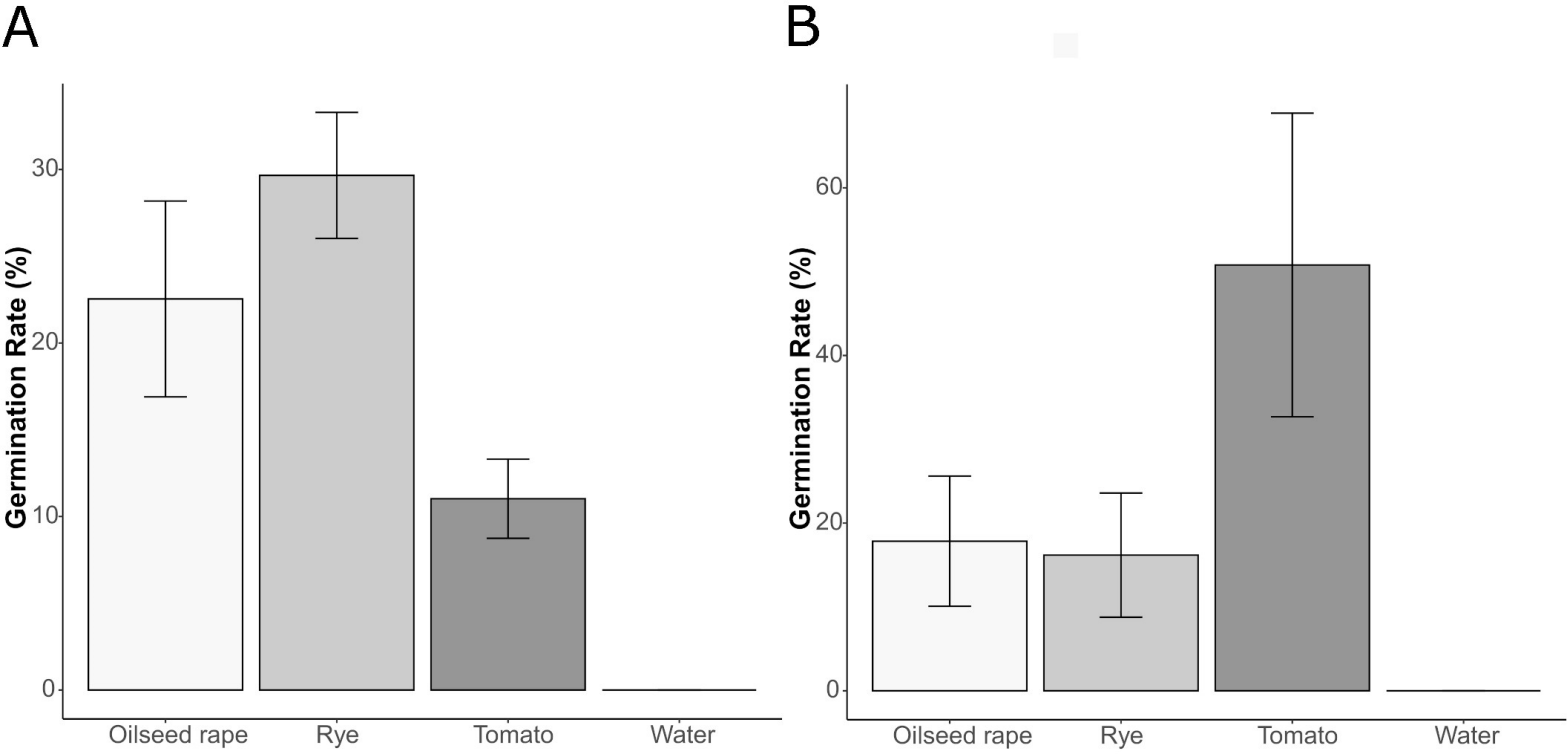
Effect of root exudates versus water control on the germination of microsclerotia of *V. longisporum* suppressed by bacteria. (A) SB1 (4 × 10^9^ CFU/mL) and (B) *Bacillus subtilis* (4 × 10^8^ CFU/mL) were diluted eight times with different root exudates or water. Error bars indicate standard error of the mean (*n*  =  3).

Microsclerotia were cultivated in bacterial suspensions, which were subsequently diluted either with water or root exudates (10 plants/mL), from different host and non-host plants, namely oilseed rape, tomato, and ryegrass. The results indicated that microsclerotia remained in a dormant state when bacterial suspensions were diluted with water up to a ratio of 1:8. In a marked contrast, the dormancy of microsclerotia was disrupted when bacterial suspensions were diluted with root exudates of all plant species at the ratio of 1:8 (Fig. 1). To attain a more comprehensive insight into the impact of varying root exudate concentrations on germination stimulation, microsclerotia were co-cultured with one distinct bacterial concentration and varying concentrations of root exudates. Intriguingly, our results unveiled a non-linear relationship between root exudate concentration and germination stimulation. For treatments involving oilseed rape and tomato, the strongest germination-stimulating effect was not observed at the highest concentrations but at the concentrations of ½ and 1 plant/mL representing native concentrations of root exudates. Remarkably, in the absence of bacteria, germination of microsclerotia was partially or totally inhibited at 2 days after treatment (2 DAT) when cultured with higher concentrations of root exudates (Table 1).

**TABLE 1:** Effect of plant root exudates on the germination of microsclerotia of *V. longisporum* suppressed by bacteria^*a*^

Treatment	Microsclerotia germination (%)					
	Oilseed rape		Tomato		Ryegrass	
	+ Bacteria	− Bacteria	+ Bacteria	− Bacteria	+ Bacteria	− Bacteria
40× concentrated	17.8 ± 7.2ab	70.1 ± 5.9b	0.0b	15.0 ± 6.7b	87.8 ± 15.8a	0.0b
10× concentrated	11.3 ± 4.7ab	100a	0.0b	95.2 ± 8.2a	13.5 ± 7.5b	100a
Original concentration	13.7 ± 9.1ab	97.8±3.8a	44.0±14.1a	97.4±4.4a	23.3 ± 1.9b	100a
2× diluted	29.7 ± 18.4a	100a	3.4 ± 3.2b	100a	17.7 ± 11.2b	96.3 ± 6.4a
10× diluted	0.0b	97.2±4.8a	0.0b	98.7±2.2a	33.2 ± 26.5b	100a
No root exudates	0.0b	100a	0.0b	100a	0.0b	100a

^*a*^ Original concentration refers to root exudates from 1 plant/mL. “+ Bacteria" indicates presense of soil bacteria 1; " − Bacteria" indicates absence of soil bacteria 1. Different letters indicate significant differences (*P* < 0.005; Tukey's test) between treatments (*n* = 3).

### *In vitro* bioassays with polar and non-polar fractions of root exudates

To identify the specific compounds within root exudates, which are responsible for the germination-stimulating effect, root exudates of oilseed rape were separated into polar and non-polar fractions (non-polar group 1 to non-polar group 7, Fig. S1) based on their polarity. The impact of both polar and non-polar compounds on microsclerotia germination was then individually assessed. Notably, the results demonstrated that polar compounds rescued microsclerotia from bacterial suppression, thereby triggering their germination (Table 2). Regarding the non-polar compounds, the experiments revealed that while groups 1 and 2 displayed a certain level of germination-stimulating effect on suppressed microsclerotia (Table 2), the remaining groups did not exhibit a similar effect (data not shown). Furthermore, consistent with the results of bioassays conducted with root exudates, it was observed that in the absence of bacteria, microsclerotia germination was partially inhibited at 2 DAT when exposed to higher concentrations of the tested compounds (Table 2).

**TABLE 2:** Effect of polar and non-polar fractions of oilseed rape root exudates on the germination of microsclerotia of *V. longisporum* suppressed by bacteria^*a*^

	Microsclerotia germination (%)					
	Polar		Non-polar-1		Non-polar-2	
Treatment	+ Bacteria	− Bacteria	+ Bacteria	− Bacteria	+ Bacteria	− Bacteria
3 mg/mL	27.3 ± 11.4a	16.0 ± 4.1b	13.5 ± 2.0a	77.8 ± 25.5a	N.A.	N.A.
1.5 mg/mL	19.5 ± 12.5ab	30.5 ± 22.3b	4.0 ± 4.0b	78.1 ± 19.1a	16.0 ± 6.0a	49.2 ± 6.4b
300 µg/mL	4.9 ± 5.7ab	93.5 ± 11.2a	0b	94.6 ± 9.4a	19.0 ± 7.8a	83.6 ± 14.2a
3 µg/mL	1.7 ± 2.9b	100a	0b	100a	6.7 ± 6.7ab	100a
1.5 µg/mL	2.0 ± 3.5b	100^a^	0b	100a	0b	100a
300 ng/mL	0b	100a	0b	100a	0b	100a
No root exudates	0b	100a	0b	98.3 ± 2.9a	0b	100a

^*a*^ “+ Bacteria” denotes presence of SB1; “− Bacteria” denotes absence of bacteria. Different letters indicate significant differences (*P* < 0.005; Tukey's test) between treatments (*n* = 3).

### GC-MS analysis of the polar fraction of root exudates

Polar compounds were extracted from oilseed rape root exudates and subjected to gas chromatography-mass spectrometry (GC-MS) analysis. A total of 41 compounds were detected, including 11 sugars and their derivatives, 11 amino acids, 14 other carboxylic acids, 3 sugar alcohols, and 2 additional compounds that do not fall within these 4 categorized groups (Table 3; Table S1).

**TABLE 3:** Polar metabolites detected in oilseed rape root exudates by GC-MS analysis

Chemicals	Average Rt (min)	Measured retention index (RI)	Reference RI	RI similarity %
Sugars and derivatives				
Xylose	23.904	1,676.05	1,676.89	99.95
Lyxose	24.106	1,685.56	1,682.54	99.82
Arabinose	24.244	1,691.71	1,696.3	99.73
Rhamnose	25.541	1,753.48	1,754.62	99.94
Sorbose	28.662	1,907.92	1,911.49	99.81
Fructose	28.849	1,917.72	1,917.48	99.99
Galactose	29.022	1,926.84	1,927.46	99.97
Glucose	29.491	1,951.53	1,944.28	99.63
N-Acetyl glucosamine	31.648	2,053.8	2,058.7	99.76
N-Acetyl galactosamine	33.66	2,141.39	2,142.97	99.93
Sucrose	41.744	2,493.35	2,492.5	99.97
Amino acids				
Alanine	10.311	11,22.85	1,122	99.92
Valine	13.143	1,228.75	1,221.7	99.42
Isoleucine	14.558	1,284.76	1,286.7	99.85
Proline	15.176	1,308.31	1,306.38	99.85
Glycine	15.42	1,317.92	1,316.4	99.88
Serine	16.856	1,374.53	1,370.14	99.68
Threonine	17.565	1,402.76	1,396.08	99.52
Phenylalanine	23.113	1,640.07	1,642.75	99.84
Glutamine	26.302	1,789.65	1,785.52	99.77
Lysine	29.259	1,939.41	1,939.94	99.97
Glutamic acid	23.014	1,635.72	1,628.11	99.53
Carboxylic acids				
Benzoic acid	13.861	1,257.67	1,251.31	99.49
Nicotinic acid	15.019	1,302.07	1,300.8	99.90
Maleic acid	15.36	1,315.32	1,307.84	99.43
Succinic acid	15.549	1,323.01	1,315.89	99.46
Glyceric acid	16.121	1,345.6	1,339.26	99.53
Malic acid	20.016	1,503.98	1,496.36	99.49
Pyroglutamic acid	20.747	1,535.67	1,534.34	99.91
Threonic acid	21.857	1,583.75	1,574.79	99.43
Arabinonic acid	26.427	1,795.61	1,801.31	99.68
Citric acid	27.407	1,844.54	1,853.9	99.50
Quinic acid	28.353	1,892.04	1,892.02	100.00
Galacturonic acid	30.07	1,981.98	1,982.24	99.99
Saccharic acid	31.917	2,065.53	2,062.23	99.84
Oxalic acid	10.399	1,125.21	1,124.58	99.94
Sugar alcohols				
Glycerol	14.671	1,288.66	1,280.81	99.39
Erythritol	20.624	1,530.33	1,524.25	99.60
Mannitol	29.875	1,971.71	1,968.7	99.85
Others				
Uracil	16.255	1,350.9	1,344.43	99.52
Urea	13.644	1,249.36	1,246.23	99.75

### *In vitro* bioassays with AREs and individual compounds

**FIG 2:**
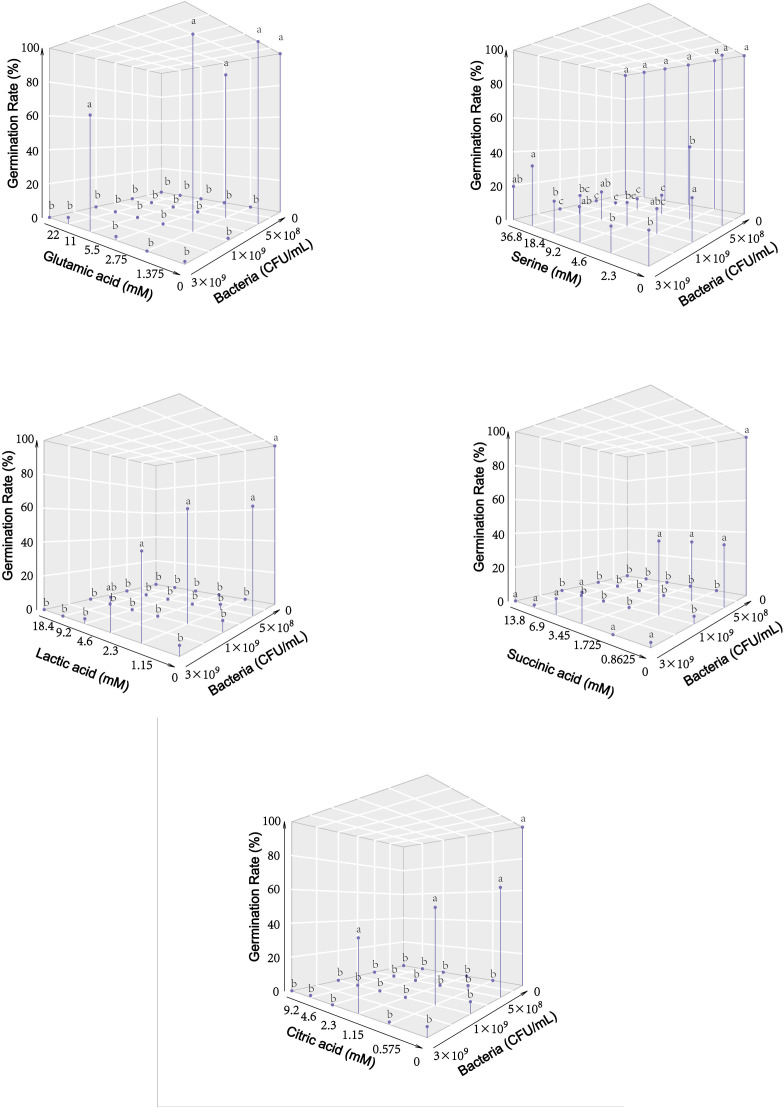
Effect of different individual compounds from the polar fractions of root exudates on microsclerotia germination. Different letters indicate significant difference (*P*  <  0.05; Tukey’s test) in germination rate among solution concentrations within the same bacterial level (*n*  =  3).

To delve deeper into the role and identity of polar compounds triggering microsclerotia germination, tests were conducted using two distinct types of artificial root exudates (AREs). These solutions consisted of common polar root exudate compounds, albeit at varying ratios (Table 5). Notably, the results revealed that ARE2 effectively stimulated the germination of microsclerotia, while ARE1 did not (data not shown). For a more precise identification of the relevant compounds, we selected specific compounds based on the AREs composition and polar compound profile obtained from GC-MS analysis. Among the 10 compounds tested, 5 demonstrated germination-stimulating effects on suppressed microsclerotia. The stimulating effect of each compound largely depends on its working concentration as well as the concentration of bacteria, under which the microsclerotia were suppressed. The highest stimulated germination rates were 100% for glutamic acid, 33% for serine, 58% for lactic acid, 38% for succinic acid, and 49% for citric acid (Fig. 2).

### Glutamic acid enhanced the germination-stimulating effect of root exudates

Different concentrations of glutamic acid were added to oilseed rape root exudates (1 plant/mL), and their respective abilities to stimulate the germination of microsclerotia were compared with root exudate treatment alone. The results indicated that glutamic acid enhanced the germination-promoting effects of root exudates on suppressed microsclerotia. However, the stimulating effect did not increase proportionally with the concentration of glutamic acid. Specifically, the germination-stimulating effect of root exudates is only enhanced when 1.38 mM glutamic acid is added. Conversely, the addition of other concentrations of glutamic acid led to a reduction in the germination rate compared to root exudates alone. For example, while the germination rate of suppressed microsclerotia with root exudate treatment alone was 17.6%, and with 5.5 mM glutamic acid treatment alone was 28.1%, the mixture of both (root exudates +5.5 mM glutamic acid) failed to induce any germination (Table 4).

**TABLE 4:** Germination rate of microsclerotia of *V. longisporum* exposed to root exudates and glutamic acid^*a*^

	Microsclerotia germination (%)									
	Water	5.5 mM Glu	2.75 mM Glu	1.38 mM Glu	0.69 mM Glu	RE	RE +5.5 mM Glu	RE +2.75 mM Glu	RE +1.38 mM Glu	RE +0.69 mM Glu
+ Bacteria	0	28.1 ± 13.0	31.5 ± 1.7	1.67 ± 2.9	0	17.6 ± 2.1	0	9.16 ± 6.0	53.0 ± 21.5	15.2 ± 8.2
− Bacteria	100	0	0	0	0	98.2 ± 3.0	0	0	0	0

^*a*^ “+ Bacteria” denotes the presence of SB1; “− Bacteria” denotes absence of SB1. RE, oilseed rape root exudates; Glu, glutamic acid. Numbers indicate the average germination rate of microsclerotia (*n* = 3).

### Rescue effect of root exudates on suppressed microsclerotia

#### Bacterial viability test

**FIG 3:**
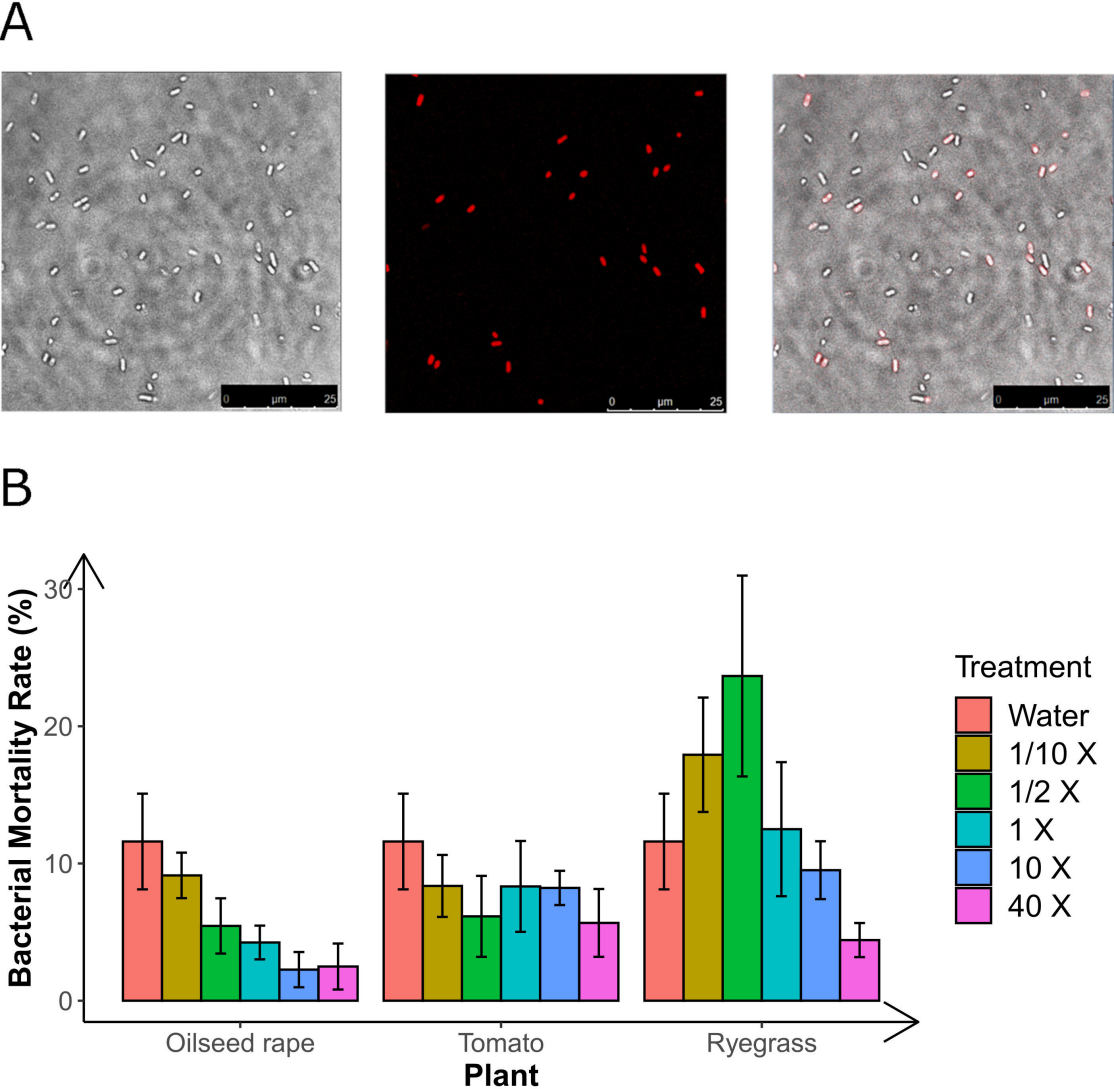
Impact of root exudates on bacterial viability. (A) Light microscopic view of bacterial cells (left), non-viable bacterial cells indicated by PI staining (center), and merged images (right). The scale bars indicate 25 µm. (B) No significant differences in mortality (*P* < 0.05) in bacterial viability were detected after treatment with water and different concentrations of root exudates (Tukey test, *n* = 3). “1/10×” to “40×” indicates the concentration of root exudates shown in Table 1.

The assessment of bacterial mortality rates in various solutions was conducted by staining the bacteria with propidium iodide (PI) and subsequently observing them through Confocal laser scanning microscopy (CLSM). Following PI staining, the nuclei of deceased spores were rendered red. In the merged image, viable bacterial cells were depicted as white spores, whereas the red color represented cells that were no longer viable (Fig. 3A). The results demonstrated no significant variations in bacterial mortality when exposed to different concentrations of root exudates or water (Fig. 3B).

#### GC-MS quantification of bacterial volatile fatty acids

**FIG 4:**
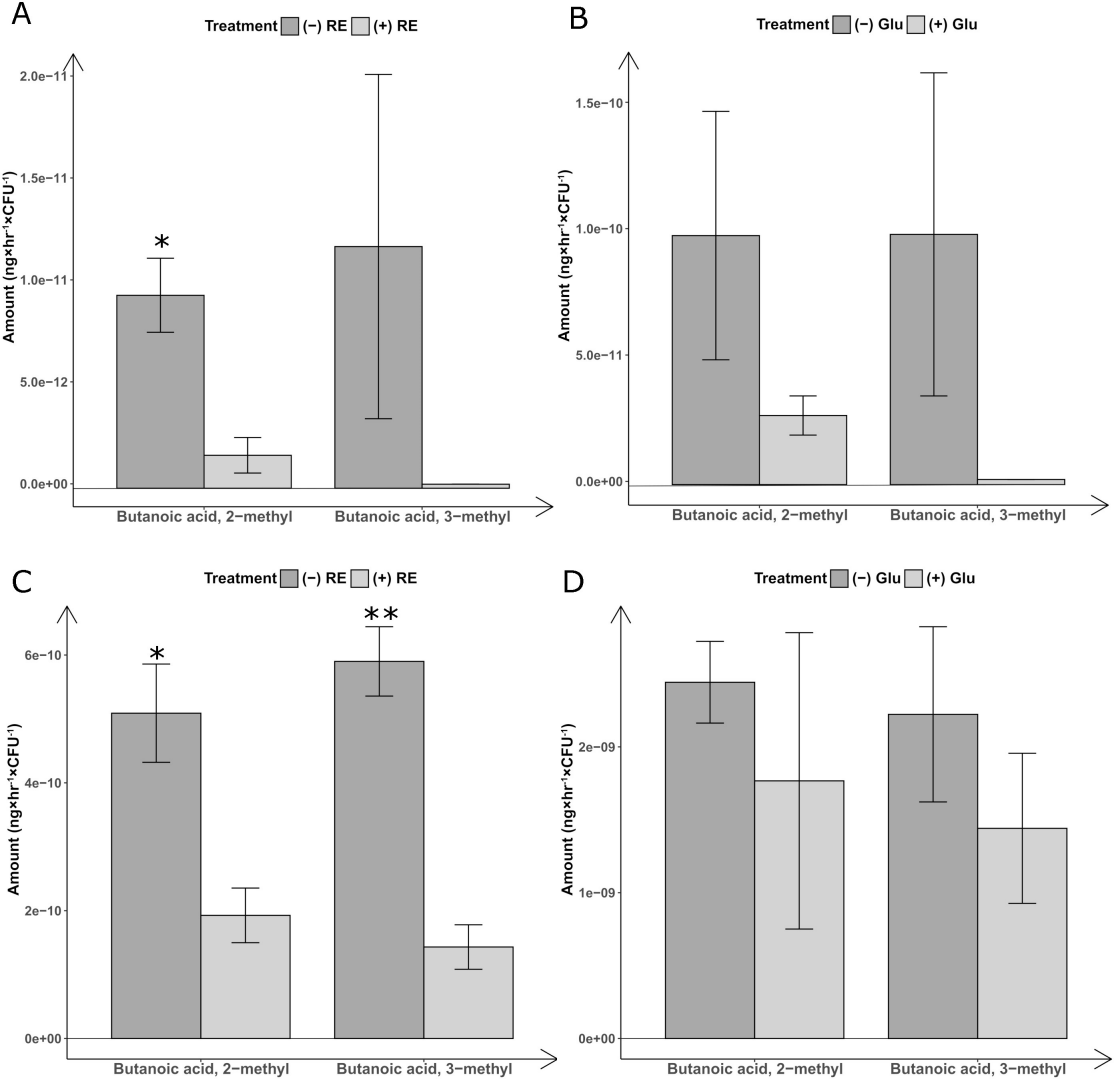
The rescuing effect of root exudates or glutamic acid on microsclerotia is associated with decreased levels of volatile fatty acids emitted by bacteria. “+ RE” denotes bacterial suspension with added root exudates and “− RE” denotes no root exudates were added to bacterial suspension. “+ Glu” denotes that glutamic acid was added to bacterial suspension and in “− Glu”, no glutamic acid was added. Significance levels are indicated by asterisks: * *P* < .05, and ** *P <* .001 (*t*-test, *n*  =  4). (A) SB1 and root exudates (*t*-test, *n* = 4) and (B) SB1 and glutamic acid (*t*-test. *n* = 3). (C) *B. subtilis* and root exudates (*t*-test, *n* = 3) and (D) *B. subtilis* and glutamic acid (*t*-test, *n* = 3).

Bacterial volatile fatty acid levels were compared among the following conditions: (i) bacterial suspension with added root exudates versus bacterial suspension without root exudates, and (ii) bacterial suspension with added glutamic acid versus bacterial suspension without glutamic acid. The results revealed a significant reduction in the production of volatile fatty acids by SB1 and *B. subtilis* upon the addition of root exudates (Fig. 4A and C). A reduction was also observed in the presence of glutamic acid; however, it did not reach the level of statistical significance for 2-methyl butanoic acid in SB1 and for both 2-methyl butanoic acid and 3-methyl butanoic acid in *B. subtilis* (Fig. 4B and D).

## DISCUSSION

As a well-known principle, soil fungistasis keeps fungal propagules like microsclerotia of *V. longisporum* dormant in the soil until plants are planted in the field and partially or entirely set off this suppressive effect in the soil (13, 14). Some earlier studies collectively affirmed the capacity of root exudates to stimulate the germination of microsclerotia when suppressed by unsterile soil conditions. For instance, Schreiber and Green (15) demonstrated that root exudates from both a host plant, tomato, and a non-host, wheat, stimulated the germination of microsclerotia of *Verticillium albo-atrum*, whose germination was suppressed by unsterile soil through an agar-disc technique. Building on these findings, later study in root observation boxes showed that root exudates from both host plants such as potato and field bean, and non-hosts like barley, can induce germination of *V. dahliae* microsclerotia, with host crops exhibiting a stronger stimulatory effect (16). Besides, this comprehensive luring effect of crop root exudates on microsclerotia germination was confirmed in both controlled laboratory and field conditions (17). These findings collectively underscore the stimulatory potential of root exudates from various plant species, regardless of their host or non-host status, in counteracting microsclerotia suppression within an unsterile soil environment. Drawing on the insights gleaned from prior studies, our current investigation seeks a fundamental understanding of the impact of root exudates on microsclerotia germination. In light of our previous research (3), which pinpointed SB as the germination-inhibiting factor in unsterile soils, our present study takes a focused approach by utilizing SB as the inhibitory agent, circumventing the complexity of unsterile soil. In this study, microsclerotia were cultivated in bacterial suspensions that were diluted with either water or root exudates from various plant species. Notably, our findings underscored that while microsclerotia remained dormant when subjected to bacterial suspensions diluted with water, a pivotal transition from dormancy to germination was induced when the same suspensions were diluted with root exudates from both host and non-host plants (Fig. 1).

To unravel the intricate relationship between root exudates concentrations and their germination stimulating effect on microsclerotia, we co-cultured suppressed microsclerotia with root exudates at a range of concentrations. Notably, our results displayed a non-linear relation between root exudate concentration and germination enhancement (Table 1), which is intriguing and may reflect the complexity of interactions in natural soil ecosystems. In natural soil ecosystems, the distribution of root exudates is influenced by a multitude of factors, including soil type, plant health, microbial activity, and physico-chemical conditions. These factors can create variations in root exudate composition and concentration over space and time. As a result, the concentration of root exudates around microsclerotia in the field may not follow a linear pattern, unlike controlled laboratory conditions. Microsclerotia, as survival structures, may have evolved mechanisms to respond to a range of root exudate concentrations, reflecting their adaptation to diverse soil conditions. Moreover, in their paper, Mol and van Riessen (16) also pointed out that the relationship should be non-linear if the concentration of root exudates determines the stimulation of microsclerotia germination. The non-linear response observed in the interaction between microsclerotia germination and root exudate concentrations might imply a trade-off strategy, where microsclerotia exhibit variable germination patterns to optimize their chances of encountering host plants suitable for infection.

In order to delve deeper into the germination stimulating effect of root exudates on microsclerotia, we fractionated root exudates into polar and non-polar fractions, encompassing primary and secondary metabolites, and tested their effect on microsclerotia germination. The result showed that fractions consisting of primary metabolites have stronger stimulating effect on germination of suppressed microsclerotia than fractions containing non-polar fractions (Table 2; Table S2). From the seven tested groups of non-polar compounds, only groups 1 and 2 showed some stimulating effect, but not as strong as polar compound fractions (Table 2; Table S2; Fig. S1). The importance of polar compounds within root exudates in triggering the germination of microsclerotia was also confirmed in the present study using AREs, encompassing sugars, amino acids, and organic acids commonly present in natural root exudates (6, 8, 18). Two AREs, ARE1 and ARE2, consisting of the same components but mixed at different concentrations were applied. Our findings showed that while microsclerotia remained dormant when subjected to bacterial suspensions diluted with water or ARE1, a pivotal transition from dormancy to germination was induced when the same suspensions were diluted with ARE2. These results offer a plausible explanation for our earlier observation, that germination of microsclerotia was stimulated by root exudates of both host and non-host plants, as primary metabolites universally exist in root exudates of plants. Unlike bioactive secondary compounds that are actively exuded by plants from their roots, primary metabolites are believed to be primarily influenced by diffusion, representing a form of “passive release” from the root (19, 20). The observed stimulatory effect of primary metabolites on the germination of microsclerotia presents an intriguing perspective on the co-evolutionary dynamics between soil-borne pathogens and their plant hosts. Primary metabolites, being passively secreted by plant roots, represent a ubiquitous and essential component of root exudates. Pathogens, over evolutionary time scales, may have developed the ability to exploit these primary metabolites as a reliable cue for germination, as they are less subject to active regulation by plants. This adaptational strategy could confer a competitive advantage to the pathogen, allowing it to efficiently respond to the presence of potential hosts in the rhizosphere. Moreover, studies have reported significant changes in primary metabolites within root exudates in response to pathogen interactions. Li et al. (10) found that the content of amino acids and sugars in root exudates is higher in the susceptible cultivar than in the moderately cultivar when facing *F. oxysporum* and *F. solani*. Similarly, research conducted by Balendres et al. (21) found that specific compounds in root exudates, such as amino acids, sugars, and organic acids, stimulate the germination of *Spongospora subterranea* resting spores in potatoes, highlighting the role of primary metabolites in pathogen germination and possibly explaining non-host specific stimulation. Additionally, they found some sugar compounds were higher in the root exudates of susceptible cultivars.

To identify compounds with potential germination-stimulating effects, we conducted bioassays with individual compounds to assess their impact on microsclerotia suppressed by bacteria. As per the composition of AREs (Table 5) and the results of GC-MS analysis of the root exudate polar fraction (Table 3), we selected nine candidate compounds: three sugars, three amino acids, and three organic acids. Additionally, one compound, pyroglutamic acid, was included because pyroglutamic acid is a derivative of glutamic acid or glutamine (22). Despite containing the same components, the AREs ARE1 and ARE2 had different concentrations of these compounds, leading to distinct effects on the germination of microsclerotia. Therefore, in this bioassay, five levels of concentration for each compound were applied. The observed germination-stimulating effects of glutamic acid, serine, citric acid, lactic acid, and succinic acid on suppressed microsclerotia highlight their potential role as crucial compounds in the stimulation of dormant microsclerotia. Notably, glutamic acid exhibited the strongest stimulating effect among these compounds (Fig. 2). In order to substantiate the role of glutamic acid in the germination-stimulating impact of root exudates, we conducted bioassays involving the cultivation of germination-suppressed microsclerotia with root exudates, glutamic acid, and a combination of both. The findings affirmed that glutamic acid enhances the germination-stimulating effect of root exudates, although the enhancement does not follow a linear pattern (Table 4). There is not much research available on glutamic acid in inducing germination of propagules of soil-borne fungi. In 1958, Stover (23) found that glutamic acid effectively induced the germination of *F. oxysporum* f. sp. *cubense* conidia in agar amended with unsterile soil. Additionally, a study by Wu et al. (24) investigating upland cotton root exudates and their impact on *V. dahliae* revealed distinct differences in amino acid compositions of resistant and susceptible cultivars. Root exudates from resistant cultivars lacked several amino acids, including glutamic acid, which were present in the root exudates of susceptible counterparts. Moreover, the relevance of organic acids, another key component in root exudates, has been exemplified in a study by Saleh et al. (25). Their research on *Brachypodium distachyon* root exudates demonstrated that organic acids play a pivotal role in shaping interactions between plants and endophytic bacteria. Specifically, these organic acids were found to influence bacterial chemotaxis and biofilm formation, indicating their multifaceted roles in plant-microbe associations.

**TABLE 5:** Composition of two AREs (18)

	Glucose (mM)	Fructose (mM)	Sucrose (mM)	Citric acid (mM)	Lactic acid (mM)	Succinic acid (mM)	Alanine (mM)	Serine (mM)	Glutamic acid (mM)
ARE1 (C/N 20.5)	18.4	18.4	9.2	9.2	18.4	13.8	9.2	9.2	5.5
ARE2 (C/N 40.1)	18.4	18.4	9.2	4.6	9.2	6.9	18.4	18.4	11

In the above mentioned bioassay with individual candidate compounds, we also investigated the interplay between bacterial concentrations and the tested solutions by evaluating various combinations of concentration levels. Notably, our findings revealed intriguing variations in the rescuing effect based on distinct bacterial concentrations. For instance, when microsclerotia were suppressed by 3 × 10^9^ CFU/mL bacteria, glutamic acid demonstrated a germination-stimulating effect at a concentration of 5.5 mM. In contrast, under the influence of 1 × 10^9^ CFU/mL bacteria, microsclerotia exhibited germination at a concentration of 1.375 mM (Fig. 2). This finding raises an intriguing question on the underlying mechanism of the rescuing effect observed in the microsclerotia germination process. It is possible that the higher bacterial concentration (3 × 10^9^ CFU/mL) creates a more intense microbial impact, where a greater concentration of glutamic acid is required to effectively counteract the bacterial inhibition and stimulate germination. Conversely, under lower bacterial concentrations (1 × 10^9^ CFU/mL), the microbial pressure on microsclerotia is less pronounced, allowing for germination at a lower concentration of glutamic acid. In this scenario, the rescuing effect of glutamic acid or other germination-stimulating compounds might involve a mechanism that directly interacts with the bacteria, potentially altering their suppressive activity and thereby influencing microsclerotia germination.

To further investigate the specific nature of the interaction between the germination-stimulating solutions and bacteria, we first checked the effect of root exudates on bacterial viability. The results revealed no significant differences in bacterial mortality rate between treatment with root exudates and water (Fig. 3). In addition, in the rye grass treatment, the highest germination rate of microsclerotia was observed at the 40× concentration (87.8%), where bacterial mortality was the lowest (4.4%). Conversely, treatments with the highest bacterial mortality—1/2× (23.7%) and 1/10× (17.9%)—recorded lower germination rates of 17.7% and 33.2%, respectively (Fig. 3; Table 1). These observations reject our hypothesis that the germination-stimulating solutions function by reducing bacterial viability subsequently promoting microsclerotia germination close to the roots. Subsequently, we speculated that root exudates might induce alterations in bacterial metabolites, particularly those possessing a potential inhibiting effect on microsclerotia. Building on our previous study, which implicated volatile fatty acids as major inhibitors to microsclerotia germination (3), we proceeded to examine the impact of root exudates and glutamic acid on the production of bacterial volatile fatty acids. Interestingly, the results showed a significant reduction in the levels of two fatty acid compounds produced by SB1 and *B. subtilis* in the presence of root exudates (Fig. 4A and C), which had been earlier identified as inhibitors of microsclerotia germination. While a reduction was also observed in the presence of glutamic acid, it did not reach statistical significance except 3-methyl, butanoic acid of SB1 (Fig. 4B and D). These compelling findings suggest that root exudates could potentially facilitate microsclerotia germination by modulating the inhibitory factors generated by SB. In addition, when culturing microsclerotia with root exudates and water, as compared to water alone, root exudates showed no significant difference in bacterial mortality and even possibly an increase in bacterial viability (Fig. 3). This suggests that root exudates are not an adverse condition for bacteria and may even be advantageous. The phenomenon of bacteria reducing their production of fatty acids in favorable conditions can be explained by their versatile metabolic capabilities, which allow them to switch to alternative pathways to avoid unnecessary energy expenditure.

**FIG 5:**
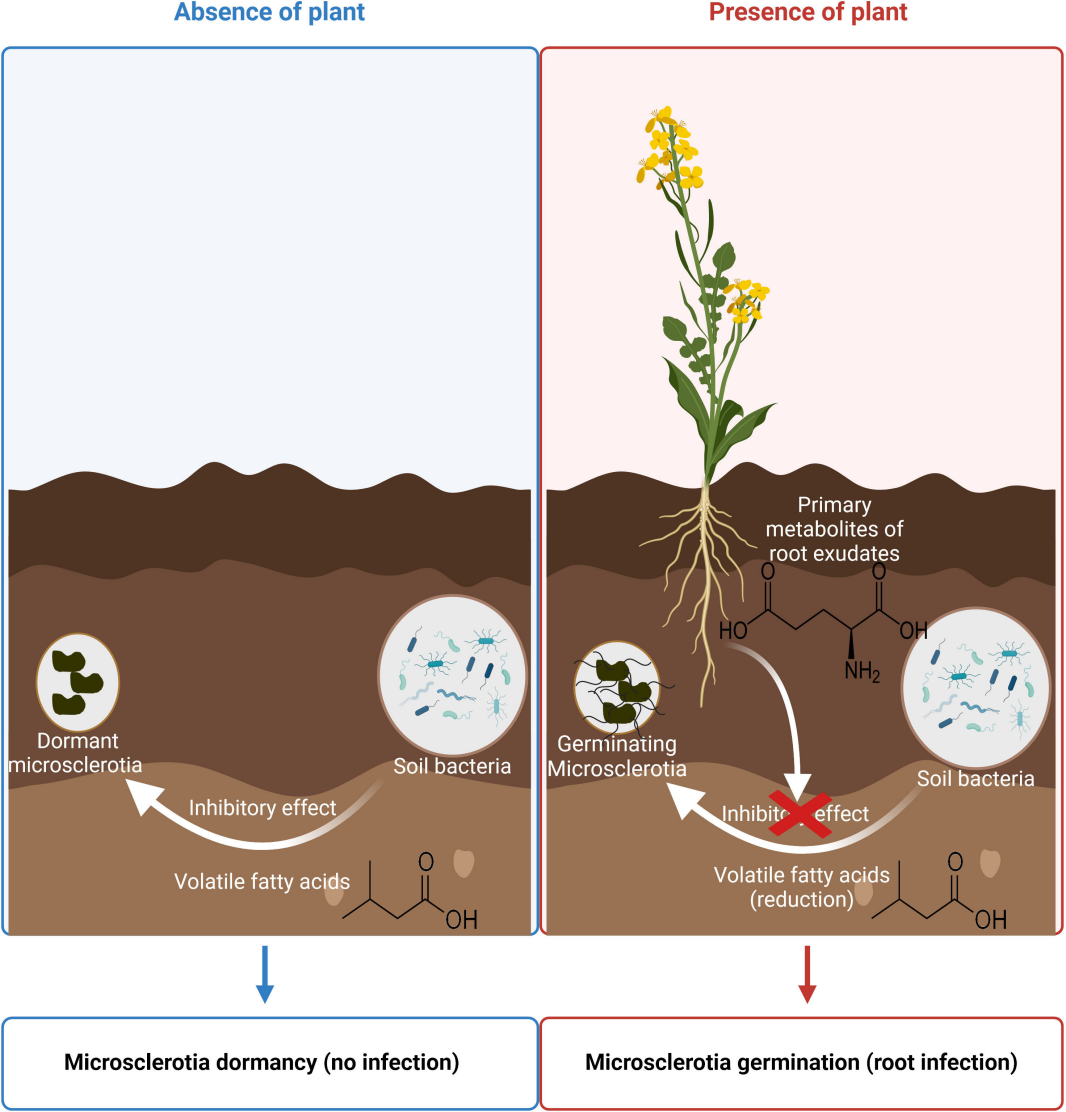
Schematic overview on how root exudates counteract bacterial suppression and induce germination of microsclerotia. Our previous study (3) identified volatile fatty acids as the main inhibitors of *V. longisporum* microsclerotia germination. The pivotal findings of this study highlight that primary metabolites in root exudates, notably glutamic acid, can suppress emission of these bacterial volatile fatty acids, thereby promoting the germination of microsclerotia. This Figure is created with BioRender.com.

Altogether, our study contributes to a deeper understanding of how root exudates and SB intricately regulate dormancy and germination of Verticillium microsclerotia in the soil ecosystem, with implications for potential novel approaches to control this soil-borne pathogen. A graphic overview has been provided to offer a comprehensive visual summary of our main findings and their significance (Fig. 5).

## MATERIALS AND METHODS

### Production of microsclerotia and cultivation of bacteria

Microsclerotia of *V. longisporum,* isolate VL43 (lineage A1/D1), were produced following the method previously described by Sarenqimuge et al. (3). Briefly, two times autoclaved mixture of 14:1 quartz sand and rye flour (with 9% of distilled water added) was prepared for producing microsclerotia. One centimeter-diameter *V. longisporum* mycelial agar plugs were punched out from edges of growing fungal colonies, placed in the sand-flour substrate (one agar plug per 225 g) and incubated at room temperature in the dark for approximately 3 weeks. After the substrate was covered with dark colored microsclerotia, it was dried in an oven (Memmert GmbH+Co. KG, Schwabach, Germany) at 25°C for approximately 1 week and passed through sieves to separate microsclerotia 100–200 µm in diameter. Prior to the bioassays, microsclerotia were assessed for potential bacterial contamination using lysogeny broth (LB) agar plates. In the event that contamination was detected, the microsclerotia were subjected to sterilization by immersing them in an antibiotic solution. The sterilization solution comprised of 50 mg/L chlortetracycline, 50 mg/L chloramphenicol, and 200 mg/L streptomycin, and the microsclerotia were allowed to stay in the solution for 2 h. Importantly, further assessments verified that this antibiotic treatment did not negatively affect the ability of microsclerotia to germinate.

Two bacterial strains were used in this study: SB1, a single strain randomly selected from a group of SB isolated from the rhizosphere of oilseed rape and filed bulk soil that demonstrated strong inhibitory effects on microsclerotia germination, and *B. subtilis* strain JH642, both reported in our previous work (3)*. B. subtilis* strain JH642 was obtained from Prof. Dr. Dieter Jahn at the Institute of Microbiology, Technical University of Braunschweig, Germany. To prepare bacterial suspensions for bioassays, a 100 µL aliquot of the bacteria stored in 25% glycerol at −80°C was incubated overnight in 6 mL of liquid LB medium. The incubation temperature was set at 25°C for SB1 and at 37°C for *B. subtilis*. The concentration of the bacterial suspension was assessed by establishing a correlation between the OD_600_ extinction (optical density of the bacterial sample measured at a wavelength of 600 nm) and the number of CFU per mL of medium.

### Preparation of plant root exudates and nutrient solutions

#### Collection of root exudates

Root exudates of rapid cycling rape (*Brassica napus*) (26), perennial ryegrass (*Lolium perenne*) and tomato (*Solanum lycopersicum*) were collected for bioassays. The seeds were first surface disinfected by soaking them in 70% ethanol at constant shaking for 10 minutes, followed by 1% NaClO for an additional 10 min, and then rinsed three times with autoclaved distilled water. Surface sterilized seeds were placed on Murashige and Skoog (MS) agar plates. To prevent plant roots from growing into the underlying MS medium, one layer of autoclaved cellophane was placed on the MS agar plate. The seeds were germinated and grown in a growth chamber at 25°C with a photoperiod of 14 h. After 12 days, seedlings were transferred to clean and autoclaved beakers filled with autoclaved double distilled water (1 seedling/mL ddH2O) and after 3 days, root exudates were harvested for bioassays. Collected root exudates were syringe filtered (0.2 µm) and lyophilized for preparing different concentrations of root exudate solutions.

#### Fractionation of root exudates

Root exudates of rapid cycling rape were prepared as described above. For this experiment, root exudates were collected after 12-day old seedlings had been transferred in autoclaved double distilled water and kept there overnight. Root exudates were concentrated to approximately 10% of its original volume using a Buchi R-100 rotary evaporator (Buchi, Flawil, Switzerland). To isolate polar metabolites, the concentrated solution was subjected to solid-phase extraction (SPE) using C18 cartridges (45 µm, 3 mL, 500 mg) from Macherey-Nagel (Düren, Germany). This SPE process was conducted on a Chromabond SPE vacuum manifold (Macherey-Nagel, Düren, Germany). The resulting filtrate, designated as the polar fraction, was then evaporated to dryness in a vacuum concentrator (Martin Christ, Osterode am Harz, Germany).

Metabolites retained on the C18 cartridges were eluted with methanol and further fractionated using a PU-2086 plus preparative high performance liquid chromatography (HPLC) system equipped with an ultraviolet–visible spectroscopy (UV/VIS) detector (UV-970) (JASCO Inc., Gross-Umstadt, Germany). The HPLC system utilized a reverse-phase Nucleodur C18 pyramid column (5 µm, 250 × 21 mm, Macherey-Nagel, Düren, Germany). The flow rate was set at 12 mL/min, with the solvent system comprising solvent A: water with 0.2% acetic acid (v/v) and solvent B: methanol with 0.2% acetic acid (v/v). The gradient elution program included an initial 5 min isocratic hold at 5% B, followed by a linear increase to 98% B over 25 min (5–30 min), a hold at 98% B for 5 min (30–35 min), a decrease to 5% B over 5 min (35–40 min), and a final re-equilibration at 5% B for 5 min (40–45 min). Monitoring was conducted at UV wavelength of 254 nm, and a total of 31 fractions were collected at 1 min intervals. Subsequently, seven fractions were carefully obtained by merging neighboring fractions, considering the UV spectra, to ensure the separation of compounds across fractions and to obtain a sufficient amount for following bioassays. The preparative chromatogram and the fractions are presented in Fig. S1.

#### Preparation of AREs and individual compounds

Two ARE solutions were prepared following Baudoin et al. (18). Three carbohydrates, three carboxylic acids, and three amino acids were mixed in two different ratios in sterilized double distilled water, resulting in two different carbon/nitrogen ratios, C/N 20.5 and C/N 40.1. The composition of each of the two AREs solutions ARE1 and ARE2 is shown in Table 1. In addition, different concentrations of several single nutrient solutions were prepared for bioassay. Those included the three carbohydrates, three carboxylic acids, and three amino acids, which the above-mentioned AREs contain (Table 5), as well as pyroglutamic acid. Concentrations of the nine single components of AREs were decided according to their concentrations in ARE2. Moreover, the concentration of pyroglutamic acid was set to be similar to that of glutamic acid. All chemicals were purchased from Sigma Aldrich, Germany.

### GC-MS analysis of root exudates

Polar fractions of root exudates were evaporated to dryness using a vacuum concentrator (Martin Christ, Osterode am Harz, Germany). Subsequently, 1 mg of each fraction was transferred into new 1.5 mL GC vials. The derivatization process, adapted from Rahman (27) and Jonsson et al. (28), involved the addition of 200 µL of methoxyamine hydrochloride (20 mg/mL in pyridine) to the samples and shaking them at room temperature for 90 min. Then, 20 µL from each sample was transferred to 200 µL glass inserts within a GC vial. For silylation, 20 µL of N-methyl-N-(trimethylsilyl) trifluoroacetamide (MSTFA) was added, and the samples were incubated for 1 h at room temperature before analysis.

The GC-MS analysis, slightly modified from Rahman (27), was conducted on an Agilent 7890B GC system coupled with a mass selective detector (MSD) (5977B) and high-efficiency source (HES) (both Agilent Technologies, Waldbronn, Germany). Chromatographic separation was achieved using an Rtx-5 GC Column (30 m, 0.25 mm ID, 0.25 µm) with a 5 m Integra-Guard Column (Restek, Bad Homburg, Germany). Each sample underwent two analyses: first with a 1:10 split ratio, then splitless, using PAL RSI 85 autosampler (PAL, Switzerland) with an injection volume of 1 µL. The temperature program was initiated at 70°C, held for 2 min, then increased to 325°C at a rate of 5 °C/min, and maintained at 325°C for 10 min. Helium was used as the carrier gas at a flow rate of 1.2 mL/min. The transfer line and ion source temperatures were maintained at 280 and 230°C, respectively. Mass spectra were acquired across a range of 70–650 m/z. GC-MS data processing was executed using MS-DIAL version 5.1.230. Metabolite identification was based on retention indices and mass spectral comparison with entries in the Fiehn Lab’s BinBase database and the Golm Metabolome Database (GMD).

### *In vitro* bioassays

#### Bioassays with collected root exudates

To investigate the effects of root exudates on microsclerotia, dried root exudates were resuspended in autoclaved double-distilled water, resulting in a concentration equal to the dried root exudates of 10 plants per mL. This solution served as the root exudates used in the bioassays. Microsclerotia were exposed to a bacterial suspension containing either 4 × 10^9^ CFU/mL of SB1 or 4 × 10^8^ CFU/mL of *B. subtilis*. Subsequently, the bacterial suspension was diluted with either root exudates or water (as control) at various ratios: 1:2, 1:4, 1:8, and 1:16. Microsclerotia germination was determined at 2 DAT with the solutions containing the bacterial suspension diluted with root exudates or water (Leica Microsystems GmbH, Germany). The experiments were conducted in microscope slides with cavities (Thermo Fisher Scientific) at 25°C in the dark.

Further investigations were conducted using 2 mL Eppendorf tubes. Three concentrations of SB1 bacterial suspension were prepared: 3 × 10^9^, 1 × 10^9^ and 5 × 10^8^ CFU/mL. These bacterial suspensions were subjected to centrifugation at 2,236 relative centrifugal force (RCF) (g force) for 5 min, and the upper solutions were discarded. The bacteria were then resuspended in autoclaved double-distilled water and washed again to remove any remaining LB medium. Then the bacteria were resuspended in different solutions of root exudates at varying concentrations, as well as in water alone. Around 30 microsclerotia were added to the 100 mL bacterial suspension mixed with either root exudates or water and were incubated at 25°C for 2 days. To determine the germination rate, all microsclerotia were observed and counted using an optical microscope (Leica Microsystems GmbH, Germany) at 2 DAT. The experiment was repeated twice with three replicates.

#### Bioassays with polar and non-polar fractions of root exudates

For the bioassay with the polar and non-polar fractions of root exudates, the dry polar fraction of root exudates was resuspended in autoclaved double-distilled water, resulting in various concentrations. A bacterial suspension of SB1 with a concentration of 3 × 10^9^ CFU/mL was prepared as described above. Next, the bacteria were resuspended in the polar fraction of root exudates at varying concentrations, as well as in water alone. Microsclerotia were added to the bacterial suspension mixed with either polar fraction or water. The suspensions were incubated at 25°C for 2 days, and the germination rates of the microsclerotia were determined. The 31 non-polar fractions were grouped into 7 groups based on their polarity (Fig. S1). To dissolve these non-polar compounds, which showed low solubility in water, 6% dimethyl sulfoxide (DMSO) was used. Separate assays confirmed that microsclerotia were able to germinate in 6% DMSO similarly to water. Bioassays were conducted as mentioned above. All bioassays were repeated twice with three replicates.

#### Bioassays with AREs and individual nutrient solutions

The bioassay with AREs and individual nutrient solutions were conducted in slides with cavities or in 2 mL tubes as described above. All bioassays were repeated twice with three replicates.

#### Impact of glutamic acid on microsclerotia germination

Lyophilized root exudates of oilseed rape were resuspended in autoclaved double-distilled water, resulting in a concentration equal to the amount of dried root exudates from one plant per mL. Varying amounts of glutamic acid were dissolved in this root exudate solution, and their ability to stimulate germination was compared with root exudates without glutamic acid. Additionally, control solutions were prepared by dissolving varying amounts of glutamic acid in autoclaved distilled water. Microsclerotia were cultured in these solutions either with or without bacteria for 2 days, and the germination rates were determined at 2 DAT.

### Bacterial viability test

To investigate whether root exudates reduced bacterial viability thus enabling germination of suppressed microsclerotia, the viability of bacteria in different treatments from section 2.3.1 was examined. PI (Carl Roth GmbH & Co., KG, Germany) at a concentration of 1 mg/mL was used to stain nucleic acids of dead bacterial cells. Bacteria suspended in root exudates or water were incubated with PI at room temperature in the dark. Fluorescence microscopy using a confocal laser scanning microscope (Leica TCS SP5, Wetzlar, Germany) was performed to visualize the PI-stained bacteria. Excitation wavelengths of 514 and 561 nm were used, and emission was recorded at 590–610 nm. The acquired images were processed using the Leica Application Suite Advanced Fluorescence software. The experiment was repeated twice with three replicates.

### Collection of bacterial VOCs and analysis by GC–MS

In order investigate whether root exudates reduce or suppress bacterial production of volatile fatty acids, bacterial volatiles were extracted and analyzed by GC-MS as mentioned above in section 2.4, following the method described previously by Sarenqimuge et al. (3). Briefly, for sampling bacterial volatile organic compounds (VOCs), 100 µL bacterial stock suspensions (SB1 concentration: 2.4 × 10^9^ CFU/mL; *B. subtilis* concentration: 3.7 × 10^8^ CFU/mL) were inoculated into 35 mL LB medium in a specifically designed 100 mL extraction vial with one inlet and one outlet. After incubation at 25°C (SB1) and at 37°C (*B. subtilis*) for 1 day, 5 mL of either root exudates (40 plants/mL, syringe sterilized with 0.2 µm filter) or autoclaved double distilled water was added to the bacteria suspension and further incubated for 2 days in the dark. LB medium added with water and root exudates served as control. Volatile collection traps containing 30 mg Porapak-Q adsorbent (Merck KGaA, Darmstadt, Germany) were attached to the outlet of the vial. Filtered and humidified air was pushed into the vial through the inlet at a rate of 0.5 L per min, while the same amount of air was pulled through the trapping filter attached to the outlet using a vacuum pump. Volatiles were collected for 3 h, and then eluted with 150 µL dichloromethane (DCM) and stored at −80°C until further analysis. In addition, the concentration of bacteria of each treatment was determined directly after extraction based on the optical density at 600 nm. This step was crucial for calibrating the quantitative results obtained from GC-MS. Compounds were quantified by comparing their peak areas to the peak area of the internal standard, tetralin (Sigma Aldrich, Germany). This experiment was also conducted using 0.2% glutamic acid (Sigma Aldrich, Germany) instead of root exudates.

### Statistical analysis

Statistical analyses were conducted using R version 4.2.2 (R Development Core Team) and RStudio Desktop version (RStudio Inc., Boston, USA). Data were analzsed using either *t*-test or one-way ANOVA (*P* < 0.05). Pairwise comparisons between groups were performed using Tukey’s honestly significant difference (HSD) test.

## Data Availability

The raw data supporting the conclusions of this article will be made available by the authors, without undue reservation.

## SUPPLEMENTAL MATERIAL

Figure S1 aem.00589-24-s0001.docx The fractionation process of non-polar compounds of oilseed rape root exudates using preparative HPLC and a modified C18 column.

Table S1 aem.00589-24-s0002.xlsx Extended table of polar metabolites detected in oilseed rape root exudates by GC-MS analysis.

Table S2 aem.00589-24-s0003.docx Second repeat examining the effect of polar and non-polar fractions of oilseed rape root exudates on the germination of microsclerotia of *Verticillium longisporum* suppressed by bacteria.

ASM does not own the copyrights to Supplemental Material that may be linked to, or accessed through, an article. The authors have granted ASM a non-exclusive, world-wide license to publish the Supplemental Material files. Please contact the corresponding author directly for reuse.
