# Supplementary material for: The interplay of suppressive soil bacteria and plant root exudates determines germination of microsclerotia of Verticillium longisporum
Source: Appl Environ Microbiol. 2024 May 30;90(6):e00589-24. doi: 10.1128/aem.00589-24 (PMC11218611; doi:10.1128/aem.00589-24)
Supplement: Figure S1 — The fractionation process of non-polar compounds of oilseed rape root exudates using preparative HPLC and a modified C18 column. [file aem.00589-24-s0001.docx]

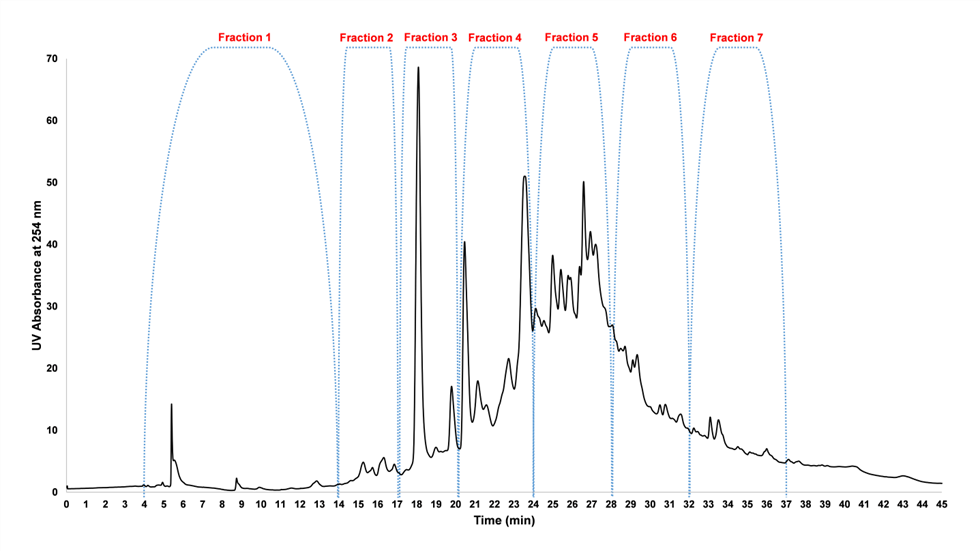


Supplementary Fig. S1. The fractionation process of non-polar compounds of oilseed rape root exudates using preparative HPLC and a modified C18 column. The 31 individual non-polar fractions were grouped into 7 sub-fractions based on their polarity.
