# Supplementary material for: The interplay of suppressive soil bacteria and plant root exudates determines germination of microsclerotia of Verticillium longisporum
Source: Appl Environ Microbiol. 2024 May 30;90(6):e00589-24. doi: 10.1128/aem.00589-24 (PMC11218611; doi:10.1128/aem.00589-24)
Supplement: Table S2 — Second repeat examining the effect of polar and non-polar fractions of oilseed rape root exudates on the germination of microsclerotia of Verticillium longisporum suppressed by bacteria. [file aem.00589-24-s0003.docx]

Supplementary Table S2. This table represents the results of a second repeat examining the effect of polar and non-polar fractions of oilseed rape root exudates on the germination of microsclerotia of *Verticillium longisporum* suppressed by bacteria.

|  | **Microsclerotia germination (%)** | | | | | |  |
| --- | --- | --- | --- | --- | --- | --- | --- |
|  | **Polar** | | **Non-polar-1** | | **Non-polar-2** | |  |
| **Treatment** | **+ Bacteria** | **- Bacteria** | **+ Bacteria** | **- Bacteria** | **+ Bacteria** | **- Bacteria** |  |
| **1.5mg/mL** | 4.6±4.1^b^ | 60.3±3.2^b^ | 20.8±4.8^a^ | 100^a^ | 26.3±1.4^a^ | 94.0±10.5^a^ |  |
| **300ug/mL** | 48.1±18.5^a^ | 100^a^ | 6.9±1.9^b^ | 100^a^ | 7.9±2.4^b^ | 100^a^ |  |
| **1.5ug/mL** | 10.0±4.8^b^ | 100^a^ | 4.4±7.7^b^ | 100^a^ | 0^c^ | 100^a^ |  |
| **300ng/mL** | 3.8±2.7^b^ | 100^a^ | 0^b^ | 100^a^ | 0^c^ | 100^a^ |  |
| **No root exudates** | 0^b^ | 100^a^ | 0^b^ | 100^a^ | 0^c^ | 100^a^ |  |

Note: “+ Bacteria” denotes the system includes soil bacteria 1 and “- Bacteria” denotes absence of bacteria. Different letters indicate significant differences (p < 0.05; Tukey’s test) between treatments; (n = 3).
